# A Real-World Data Analysis on Feline Chronic Kidney Disease in Greece: Clinical Profiles, Comorbidities, and Quality of Life

**DOI:** 10.3390/vetsci13020192

**Published:** 2026-02-15

**Authors:** Ioulia Chortara, Irene Chatzipanagiotidou, Ioli Moutsopoulou, Constantina N. Tsokana, Eleni Pavlidou, Thaisa L. Sandri, Andrea Wright, George Valiakos

**Affiliations:** 1 Asclepius One Health Platform, 10671 Athens, Greece; chortara@asclepiusoh.com (I.C.); irenechatzip@asclepiusoh.com (I.C.); moutsopoulouioli@asclepiusoh.com (I.M.); info@asclepiusoh.com (E.P.); 2 School of Veterinary Medicine, Faculty of Health Sciences, Aristotle University of Thessaloniki, 54124 Thessaloniki, Greece; ctsokana@vet.auth.gr; 3 Global Medical Affairs, Zoetis Inc., Parsippany, NJ 07054, USA; thaisa.sandri@zoetis.com (T.L.S.); andrea.wright@zoetis.com (A.W.); 4 Faculty of Veterinary Medicine, School of Health Sciences, University of Thessaly, 43100 Karditsa, Greece

**Keywords:** cat, chronic kidney disease, Greece, quality of life, real-world data

## Abstract

Feline chronic kidney disease (fCKD) is highly prevalent in geriatric cats, affecting up to 80%, and is a leading cause of morbidity in cats with a challenging diagnosis. This study aimed to characterize the clinical profiles, comorbidities, and quality of life of cats with fCKD in real-world clinical settings in Greece. Real-world data from 208 client-owned cats with fCKD were analyzed, including demographics, clinical findings, body and muscle condition scores, and practitioner-assigned stage according to International Renal Interest Society (IRIS) guidelines. Additionally, owners completed the VetMetrica™ Health-Related Quality of Life (HRQL) questionnaire. Associations between comorbidities, IRIS stage, and HRQL domains were assessed using appropriate statistical analysis. At least one comorbidity was present in 66.8% of cats, with oral/dental disease (29.81%) and anemia (15.38%) being the most common. HRQL scores declined significantly with advancing IRIS stage (*p* < 0.001). Notably, anemia showed the strongest association with reduced HRQL across all domains, including an 18.79-point reduction in Emotional Wellbeing (*p* < 0.001). Oral/dental disease was also significantly associated with poorer Comfort (OR 5.16) and Emotional Wellbeing (OR 7.10). Overall, comorbidities independently contributed to impaired quality of life in cats with fCKD. These findings highlight that early detection of fCKD and targeted management of comorbidities alongside renal care may substantially improve patient welfare.

## Simple Summary

Chronic Kidney Disease is a condition affecting cats, usually of older age, that has a severe impact on their health and daily life. In this study, more than 200 cats with CKD were investigated to evaluate their health problems and how the disease affected their wellbeing. The results showed that most cats (67%) had other health issues as well, like dental disease and anemia, and that this causes a significant reduction in the cat’s comfort and happiness. The quality of life of the animal significantly declines as the disease advances (according to the IRIS stage). These findings can help veterinarians and pet owners understand the impact of chronic kidney disease on a cat’s life, and the importance of early detection of the disease, as well as treatment of other health issues, in order to significantly improve the animal’s quality of life.

## 1. Introduction

Feline chronic kidney disease (fCKD) is among the most common and clinically significant disorders in cats, particularly in older individuals [1,2]. It represents a major cause of morbidity and mortality, with a prevalence that exceeds that reported in dogs [3,4,5]. According to the International Renal Interest Society (IRIS) [6], the prevalence of fCKD in the general feline population ranges between 1.0 and 3.0% [2,5,7,8], while in geriatric cats it may reach up to 80% [1,2,7,9].

Chronic kidney disease (CKD) is characterized by structural and/or functional damage of one or both kidneys that persists for at least three months. This irreversible loss of renal structure and/or function may remain stable for variable periods, but progresses over time [9]. Tubulointerstitial nephritis is the most commonly observed histopathologic lesion in fCKD; however, the precise underlying cause remains unclear [1,2,10]. Current evidence supports a multifactorial nature, shaped by genetic predisposition, individual susceptibility, and environmental influences [10].

Several factors increase CKD risk or contribute to its progression. Age is the most consistent risk factor [1,2,9,10,11,12], while certain breeds [1,8,13,14] and nutritional statuses [1,8,14] have also been implicated. Exposure to therapeutic or diagnostic agents [1] and the presence of comorbidities—such as hyperthyroidism, cardiovascular disease, urinary tract infections, or viral infections like FIV and FeLV—further contribute to disease progression [1,8,10,14,15].

Diagnosis is based on history, physical examination, clinicopathological data, imaging findings, and, when indicated, histopathology results [16,17]. Serum creatinine (sCr), though widely used, is insensitive in the early disease course and influenced by non-renal factors such as muscle mass, hydration, age, diet, and variation in measurement methods used [16,18,19]. More recently, symmetric dimethylarginine (SDMA) and the urine protein-to-creatinine (UP/C) ratio have been adopted as additional markers in clinical practice [16,20]. However, the diagnosis of fCKD is often delayed, usually established only once clinical signs emerge [1,16]. Low sensitivity of commonly used biomarkers, challenges in urine collection, and owners’ tendency to postpone veterinary consultation contribute to the underdiagnosis of early disease [19]. These diagnostic challenges are suggestive of the need for large-scale, real-world data to better understand how fCKD presents and progresses in a typical clinical setting.

Once a diagnosis is achieved, disease staging becomes essential for clinical management. Based on the biomarkers mentioned above, IRIS has established a four-stage grading system for fCKD. Cats are classified into IRIS stages according to sCr and/or SDMA values when they are stable and hydrated, and are further substaged based on proteinuria and blood pressure measurements [20].

Clinical signs of CKD in cats are often non-specific and may not appear until significant renal damage has occurred [1]. Polyuria and polydipsia are usually the earliest and most common manifestations [21]. As the disease advances, other signs such as weight loss, gastrointestinal disturbances, lethargy, and dehydration become increasingly common [1,22], reflecting the systemic impact of reduced renal function and the decline in overall condition.

CKD negatively affects a cat’s quality of life (QoL), leading to reduced activity, poor appetite, and difficulties with dietary modifications or medication administration. Therefore, assessing QoL provides an additional dimension for evaluating disease severity and the effectiveness of therapeutic interventions [23]. Insights from human medicine further reinforce its importance: studies consistently demonstrate that CKD reduces QoL compared to healthy individuals [24,25], with progressive deterioration in advanced stages [26].

The chronic nature of CKD also impacts owners’ mental health. Higher levels of stress, depression, and anxiety, as well as reduced QoL, have been reported among caregivers of pets with chronic or terminal conditions [27]. These findings highlight that the wellbeing of cats with CKD and their owners is closely linked, emphasizing the need to address both in clinical management and research.

In recent years, the emergence of real-world data (RWD) and real-world evidence (RWE) has created new opportunities in medical research [28]. The FDA [29] defines RWD as health-related data from sources such as medical records, insurance claims, or disease registries, while RWE is the clinical evidence generated from their analysis. In veterinary medicine, however, only a limited number of studies have incorporated RWE into routine practice [30,31]. Applying these methods to fCKD could provide new perspectives on both the clinical presentation and QoL of cats and their owners, improve disease management, and support more informed decision-making in daily practice.

This study uses a real-world data collection system for cats to comprehensively characterize the clinical profiles, comorbidities, and quality of life of cats with fCKD in Greece, representing the first multicenter real-world analysis to integrate clinician-reported data with owner-reported quality-of-life outcomes in this population. The study seeks to generate practical knowledge that can support veterinarians in diagnosis and disease management.

## 2. Materials and Methods

### 2.1. Development of Data Collection Forms

The following forms were developed to systematically collect data on fCKD cases. Their content was based on an extensive literature review in both human and veterinary studies related to kidney disease [32,33,34,35].

(a) “Screener Case Report Form” that included the specific study inclusion and exclusion criteria for fCKD cases (Table S1, Supplementary Materials) and the cat’s IRIS stage with a description of the basic guidelines for staging (Table S2, Supplementary Materials).

(b) “Veterinarian-Completed Form” to gather feline patient demographics, such as sex, age, breed, weight, Body Condition Scoring (BCS), Muscle Condition Scoring (MCS), and any comorbidities (Table S3, Supplementary Materials).

(c) “Veterinarian/Owner Completed Case Form” to be filled out by the veterinarian and the cat owner to record data about the cat’s diet, medical history, and treatment. It also included a checklist for owners to identify existing clinical signs and to report which of these they found most troublesome for themselves and their cat (Tables S4 and S5, Supplementary Materials).

(d) “Owner-Completed Form” to anonymously gather demographic data from the participating cat owners (Table S6, Supplementary Materials) who were required to be over 18 years old.

(e) “VetMetrica health-related quality of life (HRQL) Questionnaire” to evaluate the association of fCKD severity (as defined by IRIS Stages) on the cat QoL using the VetMetrica™ Feline HRQL assessment. Owners completed the questionnaire independently on the day of the consultation. This tool consists of 20 behavior-based questions answered on a 7-point Likert scale (0 = Not at all to 6 = Could not be more) [35,36,37]. This results in scores in three HRQL domains, including Vitality, Comfort, and Emotional Wellbeing. Summary scores on physical wellbeing were also calculated. For interpretability, raw domain scores (0–6) were normalized to a 0–100 scale.

### 2.2. Participant Recruitment

Recruitment was conducted by veterinarians in Greece who are part of the Asclepius One Health (AOH) network, an independent non-profit organization operating internationally from Greece [31,38]. Participating veterinarians identified eligible cats (a) by searching their clinic’s medical records for already diagnosed fCKD cases and (b) by enrolling new fCKD cases that were admitted to their clinics between October 2023 and July 2024. In both cases, owners who agreed to participate admitted their cats to the veterinary clinics and provided written informed consent during their appointments for clinical examination and blood sampling.

Most cat owners who participated in the study were female (175/208, 84.1%). Both female and male owners exhibited similar mean age (45.7 and 46.4, respectively) and age range (20–78 and 20–74, respectively). Owner demographics, such as sex and age, showed no notable patterns. Detailed demographics are included in the Supplementary Materials (Table S7, Supplementary Materials).

### 2.3. Enrolment of fCKD Cases

Eligible cats were client-owned, aged one year or older, in a stable health condition, and with a diagnosis of CKD confirmed by the responsible veterinarian at any IRIS stage.

Cats were excluded if they were pregnant, lactating, on an unbalanced diet, or had been hospitalized within seven days prior to enrollment. Further criteria for exclusion were comorbidities that could confound the assessment of fCKD, such as acute kidney injury, endocrine disorders (e.g., uncontrolled hyperparathyroidism), specific congenital or obstructive renal conditions, and suspected or confirmed malignancy. Cats recently treated for parasitic or infectious diseases known to cause kidney damage (e.g., leptospirosis) or concurrently participating in a clinical trial involving the administration of any investigational product were also excluded (Table S1, Supplementary Materials).

Practitioners were encouraged to collect serum and, when feasible, urine samples at two Timepoints [Timepoint 1 (Day 0–7 from first consultation) and Timepoint 2 (Day 30–37 from first consultation)] to assist with cCKD diagnosis and IRIS staging; they were submitted for biochemical and urine analysis, respectively. The previously mentioned data collection forms were completed at both Timepoints.

### 2.4. Laboratory Analysis

The samples were sent to a commercial veterinary laboratory in Athens, Greece, which is accredited under ISO 17025 [39] and certified with ISO 9001 [40] (Vet in Progress Plus, Attiki, Greece).

Serum biochemical parameters were analyzed using a SIEMENS Dimension EXL with an LM clinical chemistry analyzer (Siemens Healthineers, Erlangen, Germany). Electrolyte concentrations were measured using a JOKOH EX-DS electrolyte analyzer (JOKOH Co., Ltd., Kanagawa, Japan). Serum analysis included blood urea nitrogen (BUN), albumin (ALB), total proteins (TP), phosphorus (P), calcium (Ca and Ca++), potassium (K), sodium (Na), and chloride (Cl), along with the Na/K ratio. Blood creatinine (CREA) was measured using a validated enzymatic assay [41,42], for which the laboratory is accredited under ISO 17025. Urinary creatinine, urinary protein, and the urinary protein-to-creatinine ratio (UP/C) were measured in urine samples.

### 2.5. CKD Diagnosis and IRIS Staging

The final diagnosis of fCKD was based on the results of the biochemical analysis, the cat’s medical history, clinicopathological findings, and any additional diagnostic tests performed (e.g., imaging) at the discretion of the responsible veterinarian. The IRIS stage was determined by the responsible veterinarians according to IRIS guidelines [43]. Serum creatinine concentration was the primary biomarker used for staging in the majority of cases, reflecting routine clinical practice. SDMA values were incorporated into IRIS staging when available, but since they were not consistently available across the entire study population, they were not included in the analysis. Similarly, data required for substaging, such as proteinuria assessment via UP/C, were not uniformly available in the overall study population. The practitioner-assigned IRIS stage was used as a practical indicator of each cat’s disease progression in a real-world setting.

The diagnosis of all comorbidities, including anemia and hypertension, was made by the responsible veterinarian based on their clinical assessment and the diagnostic tools available at their individual practice. No standardized diagnostic protocol or specific quantitative thresholds (e.g., a specific value for systolic pressure for hypertension) were provided to the participating veterinarians, thus reflecting the real-world clinical decision-making process.

### 2.6. Statistical Analysis

Statistical analyses were conducted using IBM SPSS Statistics (Version 29.0.2.0, Armonk, NY, USA) and R (Version 2024.04.1). All statistical tests were two-tailed, and a *p*-value of <0.05 was considered statistically significant. All analyses utilized only data from Timepoint 1.

Descriptive statistics were employed to summarize demographic and clinical characteristics of the study population. Normality of continuous variables was assessed using the Kolmogorov–Smirnov or Shapiro–Wilk tests. Comorbidities were classified into categories based on the primary organ system, as detailed in Table S8 (Supplementary Materials).

Chi-square tests were applied for categorical variables. Kruskal-Wallis and Mann–Whitney U tests were used for non-parametric comparisons. One-way analysis of variance was followed by post hoc pairwise comparisons using *t*-tests with a Bonferroni correction, and *t*-tests were used for parametric comparisons of normally distributed continuous variables. A Benjamini–Hochberg correction was applied to the ANOVA results to control for multiple comparisons. Dunn’s test with a Bonferroni correction post hoc test was used for pairwise comparison when statistically significant results were recognized.

Comorbidities were analyzed as categorical (present/absent) variables. A multi-step approach was used to analyze the association of fCKD and comorbidities with HRQL scores.

First, Permutational Multivariate Analysis of Variance (PERMANOVA) was used to assess the overall association of categorical variables (like presence of comorbidity) with multidimensional VetMetrica™ scores.

Next, odds ratios (OR) with 95% confidence intervals (CI) were calculated to evaluate the likelihood of QoL deterioration associated with specific comorbidities. For this analysis, a ‘deteriorated’ HRQL was defined as a score falling below the median score of the control group (cats without any comorbidities) for that specific domain.

Finally, a multiple linear regression analysis was conducted to determine the independent relationship between the variables (specific comorbidities) and the deterioration in HRQL scores for each domain. Comorbidities in fewer than ten cats in the dataset were merged into a single “All Other” category to ensure adequate sample size and model stability for each predictor.

To evaluate the correlation between the presence of a clinical sign (binary variable) and the severity of fCKD as depicted by the IRIS Stage reported (considered in this analysis as an ordinal variable), the point-biserial correlation coefficient (*r_pb)_* was calculated. The equality of proportions of tests was conducted only for clinical signs with at least 20 cases to ensure the validity of the test.

## 3. Results

### 3.1. Enrolled fCKD Cases

A total of 42 practitioners provided data on 208 fCKD cases that were admitted to their clinics. The majority (38, 90.4%) were general practitioners, while four of the practitioners were referral clinicians. More than half of the data (119/208, 57.2%) was obtained from 11 major veterinary clinics, while 31 practitioners contributed the remaining 42.7% (89/208) of the fCKD cases. The mean number of cases contributed per clinic was five (range: 1–23).

Most of the fCKD cases (135/208, 64.9%) were classified by the participating veterinarians as IRIS Stages 1 and 2, while 35.1% (73/208) were classified as IRIS Stages 3 and 4 at Timepoint 1. Timepoint 2 measurements were completed for 177 cases following a 30-day interval, during which 24 cats died, and seven were lost during follow-up (owner moved out of town, refused second examination, etc.). The distribution of the recorded cases per IRIS Stage reported by the practitioners for Timepoints 1 and 2 is presented in detail in Table 1. No statistically significant difference was observed between the two timepoints with regard to the frequency of IRIS stages reported (chi-square = 4.36, df = 3, N = 208, *p* = 0.226).

### 3.2. fCKD Cases Demographics

Female (93/208, 44.7%) and male cats (115/208, 55.3%) were almost equally represented in the study population. Among the 208 cats enrolled, most of them were spayed/neutered (198/208, 95.2%). There was no statistically significant association between sex and neuter status (Fisher’s exact test *p* = 0.157) or practitioner-assigned IRIS stage (Fisher’s exact test *p* = 0.245) (Table 2).

Among the eight feline breeds recorded, the Domestic Shorthair (DSH) was the most common (166/208, 79.8%) (Table S9, Supplementary Materials). Βreed was not associated with practitioner-assigned IRIS stage (Fischer’s exact test *p* = 0.192).

**Figure 1:**
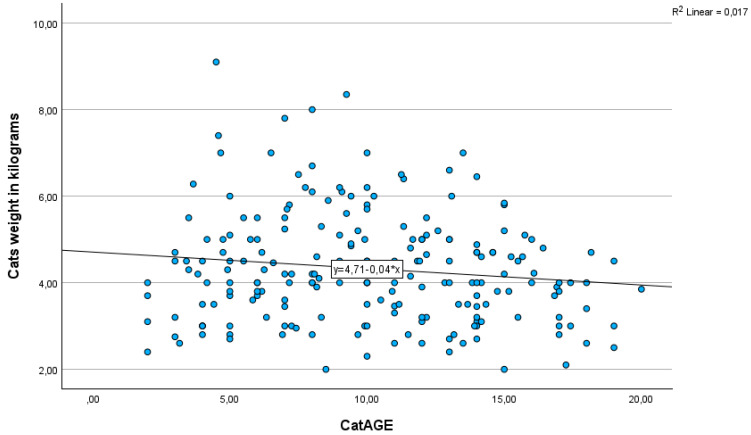
Scatter plot for feline age and weight with Pearson Correlation. The line represents the linear regression fit.

The age distribution was found to be non-normal (K-S normality test = 0.64, *p* = 0.037, Figure S1, Supplementary Materials). The median age of the cats was 10.1 years old (SD = 4.38), with a range of 2 to 20 years. There was no statistically significant difference in the age distribution across the four IRIS stages (Kruskal-Wallis, test statistic = 5.352, *p* = 0.148). The median weight of cats was 4.1 kg (SD = 1.27, range 2–9.1). Upon analysis of feline age and body weight, no statistically significant correlation was observed, although a trend of reducing feline weight as the age increased was observed (r = −0.131, *p* = 0.059) (Figure 1).

Muscle condition score (A-Normal muscle mass, B-Mild muscle loss, C-Moderate muscle loss, and D-Severe muscle loss) [44,45] was significantly associated with advanced fCKD based on the IRIS staging. More specifically, moderate (*n* = 64) to severe (*n* = 6) muscle loss was more common in late IRIS stages 3 and 4 compared to early IRIS stages 1 and 2 (33/73, 45.2% vs. 37/135 27.4%). Conversely, normal muscle mass (*n* = 37) and/or mild muscle loss (*n* = 101) were more frequently observed in early IRIS stages, with normal muscle mass present in 20.7% (28/135) of early IRIS stages versus 12.3% (9/73) of late IRIS stages, and mild muscle loss in 51.9% (70/135) versus 42.5% (31/73), respectively. The association between muscle condition score and early vs. late IRIS stages was statistically significant (chi-square = 7.20, df = 2, N = 208, *p* = 0.027).

Similarly, a poorer BCS [44,46] was significantly linked to more advanced IRIS stages. Cats classified as below the ideal BCS (1–3) were more frequent in late IRIS stages (46/73, 63.0%) than in early IRIS stages (64/135, 47.4%), while animals above the ideal BCS (7–9) were more commonly classified at the early IRIS stages (38/135, 28.1% vs. 7/73, 9.6%). For cats with an ideal BCS (scores 4–6), proportions were similar between early and late IRIS stages (33/135, 24.4% vs. 20/73, 27.4%), indicating no difference. The association between BCS and early vs. late IRIS stages was statistically significant (chi-square = 9.89, df = 2, *p* = 0.007), with the proportion of animals below the ideal BCS increasing as IRIS stage advanced.

### 3.3. Clinical Signs

The most prevalent clinical signs reported in cats with fCKD were increased water consumption (105/208, 50.5%), weight loss and muscle wasting (85/208, 40.9%), and polyuria (84/208, 40.4%). These were followed by loss of appetite (38.5%), unkept appearance (30.8%), halitosis (29.3%), and vomiting (26.4%) (Table S10, Supplementary Materials).

A positive correlation, indicating that the frequency increased with advancing IRIS stage, was found for all the clinical signs, except for vomiting, gastroenteritis, mouth ulcers, vision disorders, fragile bones and blood in urine (*p* > 0.05). Significant but weaker correlations were also found for weight loss/muscle wasting (*p* = 0.008), anorexia (*p* = 0.002), halitosis (*p* = 0.028), pale gums (*p* = 0.002), and diarrhea (*p* = 0.045) (Table S11, Supplementary Materials).

Increased water consumption, reduced appetite, unexplained weight loss, increased urination, depressed mood, and weakness/fatigue were most frequently regarded by the owner as troublesome for the cat. In contrast, signs such as halitosis and unkept appearance were considered significantly more disturbing for the owner than for the cat (Table S12, Supplementary Materials).

### 3.4. Biochemical Examinations and Urinalysis

Biochemical analysis revealed median concentrations for CREA of 2.12 mg/dL (IQR: 1.68–3.48) and for BUN of 43.00 mg/dL (IQR: 31.00–78.50). As expected for non-normally distributed data, a notable difference was observed between the mean and median values (e.g., CREA: 3.04 vs. 2.12 mg/dL), confirming a positive skew due to the presence of individuals with advanced disease. The median P concentration was 5.05 mg/dL (IQR: 4.25–6.40). For ALB, which followed a normal distribution, the mean was 2.96 ± 0.40 g/dL. Other key electrolytes with skewed distributions included K with a median of 4.40 mEq/L (IQR: 4.00–4.90) and Na with a median of 153.00 mEq/L (IQR: 148.00–155.00) (Table S13, Supplementary Materials).

As expected, based on the IRIS staging guidelines, median concentrations of CREA (S1 = 1.34, S2 = 2.00, S3 = 3.49, S4 = 5.18 mg/dL), BUN (S1 = 26, S2 = 39, S3 = 76, S4 = 168 mg/dL), and P (S1 = 4.8, S2 = 4.6, S3 = 5.8, S4 = 10.5 mg/dL) generally increased with advancing disease stage, with statistically significant differences observed between the groups (adjusted *p* < 0.001 for all). In contrast, no significant inter-stage differences were found for ALB, Ca, Ca++, TP, and Na, K, Na/K ratio, and Cl (all adjusted *p* > 0.05) (Table S14, Supplementary Materials).

Urinalysis data were available for a subset of 35 cats. The mean specific gravity (SG) was 1023.77 ± 10.16. For urine pH, the mean was 6.66 ± 0.73, and the UP/C ratio had a mean of 0.66 ± 0.69 (Table S15, Supplementary Materials).

### 3.5. Health-Related Quality of Life Measurement (VetMetrica™) and IRIS Stages

A statistically significant decline in QoL scores across practitioner-assigned IRIS stages was observed for the domains Vitality, Comfort, Emotional Wellbeing, and Physical Wellbeing (Table 3). The Kruskal-Wallis tests revealed highly significant effects of IRIS stage for Vitality (*p* < 0.001), Comfort (*p* < 0.001), Emotional Wellbeing (*p* < 0.001), and Physical Wellbeing (*p* < 0.001). Post hoc multiple comparisons showed that cats in IRIS Stage 4 had significantly lower scores compared with those in Stages 1 and 2 across all domains (adjusted *p* < 0.001 for Vitality, Comfort, Emotional Wellbeing, and Physical Wellbeing). Additional significant differences were observed between Stage 4 and Stage 3 for Vitality (adj. *p* = 0.019), Emotional Wellbeing (adj. *p* = 0.011), and Physical Wellbeing (adj. *p* = 0.026), and between Stage 3 and Stage 2 for Vitality (adj. *p* = 0.006), Comfort (adj. *p* = 0.003), Emotional Wellbeing (adj. *p* = 0.003), and Physical Wellbeing (adj. *p* = 0.002).

When comparing cats at early and late IRIS stages, a significant decline in QoL was observed across all domains (Table 4). Mann–Whitney tests revealed significantly lower median scores in cats in a late IRIS stage for Vitality (*p* < 0.001), Comfort (*p* < 0.001), Emotional Wellbeing (*p* < 0.001), and Physical Wellbeing (*p* < 0.001).

### 3.6. Comorbidities

Most of the cats (139/208, 66.8%) presented with at least one comorbidity. When diagnoses were grouped into broader disease categories, oral/dental diseases were the most prevalent, affecting 29.8% (62/208) of the cats. Other common categories included hematologic abnormalities (15.4%), infectious diseases (10.6%), and both renal/urinary tract and cardiovascular diseases (9.6%) (Table S16, Supplementary Materials). The single most frequently reported individual diagnosis was periodontal disease, which was also present in 46/208 (22.1%) cats. Other common concomitant diseases included stomatitis (16.8%), anemia (15.4%), and mild liver disease (7.2%) (Table S17, Supplementary Materials).

**Figure 2:**
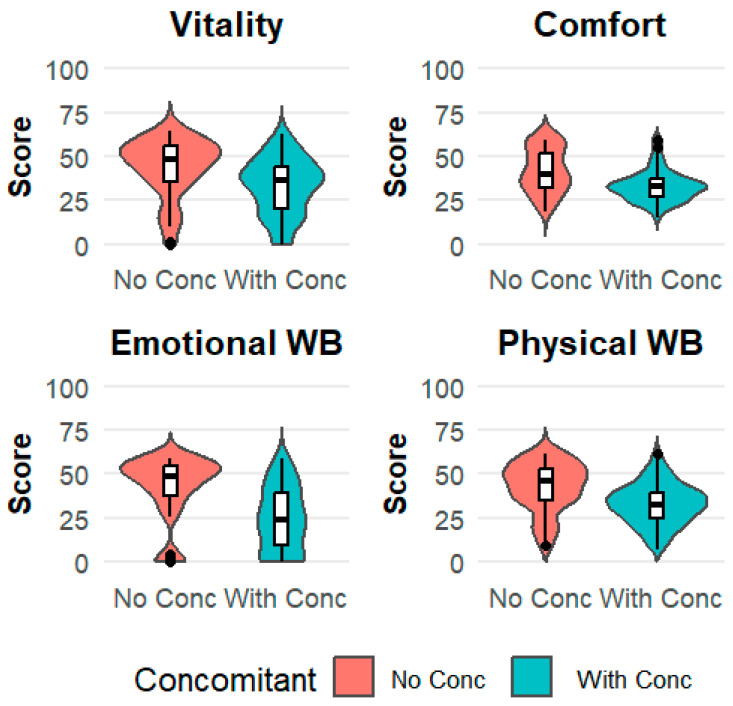
Violin plots with an overlaid boxplot of the three VetMetrica domain scores and the Physical Wellbeing score across the two groups (Cats with fCKD and no concomitant diseases vs. Cats with fCKD and concomitant diseases).

The PERMANOVA analysis revealed a significant effect of comorbidities on the combined QoL measures (*p* = 0.001) (Table S18, Supplementary Materials). Comparisons of VetMetrica QoL scores between cats with and without concomitant diseases revealed that cats with any comorbidity had significantly lower median scores across all four HRQL domains (Mann–Whitney, *p* < 0.001 for all) (Figure 2, Table 5).

To further explore the specific association of comorbidities with HRQL, an OR analysis was performed. Specifically, cats with anemia were markedly more likely to experience deterioration in HRQL, with an OR of 7.0 (95%CI: 1.62–30.33, *p* = 0.009) for the Vitality domain, 14.85 (95%CI: 1.98–111.51, *p* = 0.009) for Comfort, 12.65 (95%CI: 1.68–95.13, *p* = 0.014) for Emotional Wellbeing, and 13.0 (95%CI: 1.73–97.75, *p* = 0.013) for Physical Wellbeing. Similarly, oral/dental disease as comorbidity were significantly associated with reduced HRQL scores, with an OR of 2.55 (95%CI: 1.19–5.45, *p* = 0.016) for Vitality, 5.16 (95%CI: 2.08–12.80, *p* < 0.001) for Comfort, 7.10 (95%CI: 2.44–20.71, *p* < 0.001) for Emotional Wellbeing, and 4.43 (95%CI: 1.78–11.02, *p* = 0.001) for Physical Wellbeing (Table 6).

To control covariates, a multiple linear regression analysis confirmed that anemia, oral, and infectious diseases as comorbidities were significantly and independently associated with deterioration across multiple HRQL domains (Table 7). In the Vitality domain, anemia was associated with an almost 15-point reduction (*p* < 0.001), while oral disease corresponded to a 5.78-point decrease (*p* = 0.025). In the Comfort domain, hematologic, oral, and infectious comorbidities were linked to declines of 7.99 (*p* < 0.001), 5.09 (*p* = 0.002), and 7.77 points (*p* = 0.001), respectively. For Emotional Wellbeing, hematologic, oral, and infectious diseases were linked to reductions of 18.79 (*p* < 0.001), 7.25 (*p* = 0.012), and 9.34 points (*p* = 0.026), respectively. Finally, in the Physical Wellbeing domain, hematologic, oral, and infectious comorbidities were associated with decreases of 11.50 (*p* < 0.001), 5.44 (*p* = 0.006), and 6.11 points (*p* = 0.033), respectively.

### 3.7. Medical History and Diet

Medical history, as recorded in an open text box by the veterinarians, showed that the diagnosis in 39.9% (81/203) of fCKD cases was a result of observable signs of illness by the owner. On the other hand, in 5.4% (11/203) of the cases, CKD was discovered incidentally during routine health screenings or while investigating other medical conditions. In 30% (61/203) of the cases, the medical history was unknown. With regard to diet, commercial dry food and prescribed clinical diets were provided to a large proportion of cats (127/208, 61.1%), while smaller percentages received canned food (81/208, 38.9%), food in pouches (48/208, 23.1%), and/or homemade formulations (25/208, 12.0%).

## 4. Discussion

This study characterized the clinical presentation, HRQL, and the association of comorbidities in a large, real-world population of cats with fCKD, providing novel real-world evidence from a multicenter cohort that links comorbidities with both clinician-assessed clinical features and owner-reported HRQL. Most of the enrolled fCKD cases were in early IRIS stages, as assigned by the practitioners. The principal finding was the significant and independent negative association of comorbidities with HRQL scores across all domains. While our results confirmed the expected decline in HRQL with advancing IRIS stage, they demonstrated that concurrent diseases are significantly associated with poor QoL, emphasizing the necessity of a holistic approach to the diagnosis and management of fCKD. Our study showed the association of deteriorating physical condition, such as lower body weight and muscle loss, with advanced IRIS stages and identified a discrepancy between the clinical signs considered most troublesome for the cat versus the owner.

A critical finding in our study was the strong association between anemia and poor HRQL, with odds ratios for deterioration exceeding 12.0 for Comfort, Emotional Wellbeing, and Physical Wellbeing. This is clinically relevant, as anemia directly leads to weakness, fatigue, and reduced oxygen-carrying capacity, which would be reflected by the Vitality and Physical Wellbeing domains. Our results, therefore, build on previous observations that anemia adversely affects the quality of life of CKD patients through its overall effects [47] and contributes directly to the clinical expression of uremic syndrome in cats with advanced CKD [48].

These findings are particularly impactful given the complex nature of anemia in the context of CKD. The pathophysiology of CKD-associated anemia is multifactorial, involving reduced erythropoiesis, shortened erythrocyte lifespan, and increased erythrocyte loss [47]. While anemia is known to intensify with advancing IRIS stage due to progressive loss of renal tissue and erythropoietin production [2,49], its direct relationship with survival time is not established, with conflicting evidence across studies [12,50]. By demonstrating a negative association with QoL, our findings elevate the management of anemia in feline CKD to a primary therapeutic target.

Oral and dental diseases emerged as the most common comorbidities, affecting 29.8% of the investigated cats, consistent with previous evidence suggesting a link between oral disease and fCKD. Trevejo et al. (2018) demonstrated that cats with periodontal disease had a significantly higher risk of developing fCKD compared to those without [51]. The greatest risk was observed in cats with IRIS stage 3 or 4, indicating a severity-dependent relationship between oral and renal pathology. Similarly, Greene et al. (2014) identified periodontal disease as a clinical factor associated with increased odds of fCKD diagnosis, alongside thin body condition, weight loss, dehydration, and anesthesia within the preceding year [13]. In contrast, Vetter et al. (2023) investigated renal function in cats with histologically confirmed caudal stomatitis and found no significant difference in the frequency of kidney disease, azotemia, or proteinuria compared with age-matched controls [52]. Interestingly, cats with caudal stomatitis exhibited biochemical markers of systemic inflammation, such as elevated serum phosphorus, potassium, and globulin concentrations, along with decreased albumin and hematocrit levels, which may represent early subclinical alterations preceding fCKD.

Human medical literature mirrors these findings, demonstrating a consistent association between periodontal disease and CKD, which strengthens with disease severity [53]. Proposed mechanisms include systemic inflammation, oxidative stress, immune dysregulation, and microbial alterations, all of which can contribute to vascular and renal injuries. Although causality has not been established, human studies emphasize the need to determine whether the prevention or treatment of periodontal disease can reduce the incidence or severity of CKD, an issue that holds equal importance in feline medicine [54].

Our study supports these findings as the strong association of oral/dental disease with lower Comfort (OR = 5.16) and Emotional Wellbeing (OR = 7.10) scores indicates that the clinical burden is associated with chronic pain and distress, which directly impairs the cat’s QoL.

In our study, the presence of infectious diseases was a significant factor in patient wellbeing, showing a positive correlation with deterioration across all four HRQL domains. Infectious diseases were associated with a 9.34-point decline in Emotional Wellbeing and a 7.77-point decline in Comfort. This positions the negative association of infectious disease as intermediate between the more severe association with anemia (e.g., an 18.79-point drop in Emotional Wellbeing) and the moderate effects of oral comorbidities (e.g., a 7.25-point drop in Emotional Wellbeing).

The specific viral diseases recorded in our study population comprised FIV in 6.73%, FIP in 2.40%, and FeLV in 1.92% of cats. The pathological link is clearest with FIP, which is known to cause granulomatous nephritis [55]. While FeLV is primarily associated with neoplastic processes, a potential link to CKD has also been suggested [1,55]. The role of FIV is more controversial; however, it has been linked to the induction of renal lesions that could plausibly contribute to the decline in HRQL we observed [15,56]. Therefore, managing these infections is critical for quality of life, and this finding aligns with existing guidelines for retroviral diseases, which recommend addressing comorbidities to improve patient outcomes [57].

The systemic burden is visibly reflected in the patient’s physical condition. The present study identified a relationship between feline body composition and practitioner-assigned IRIS stage, with BCS and MCS showing signs of decline as the disease progressed. This finding validates these simple, in-clinic assessments as indicators of disease severity and the cat’s overall health status. This pattern reflects the multifactorial impact of fCKD on body composition.

Weight loss in affected cats is often gradual and may begin months before clinical diagnosis [1,8,13,22,58]. It is influenced by reduced appetite, nausea, vomiting, and diarrhea—common signs in later stages—as well as by metabolic alterations associated with chronic inflammation and decreased protein or caloric intake [59]. Over time, these mechanisms contribute to both generalized weight loss and selective muscle wasting, manifesting as sarcopenia or cachexia, depending on the underlying cause [9,58,60,61].

Given these interrelated mechanisms, weight alone provides an incomplete picture of nutritional and clinical status in cats with CKD. Incorporating BCS and MCS evaluation allows for a more accurate assessment of disease impact and progression. This combined approach is increasingly emphasized by major veterinary organizations [18,44,58,62], which recommend that each clinical visit include not only weight measurement but also assessment of body and muscle condition. Regular monitoring of these parameters enables earlier detection of nutritional deterioration [18,58] and facilitates timely intervention to preserve lean mass and improve quality of life in cats with CKD. Our study provides quantitative HRQL evidence to support this combined assessment approach. Disease progression as indicated by the practitioner-assigned IRIS stages was significantly associated with a decline in physical condition (BCS and MCS) as well as lower HRQL scores in the Physical Wellbeing and Vitality domains, indicating the systemic impact of the deterioration of physical condition on the patient’s daily life.

This study further documented that several clinical signs showed a positive correlation with disease severity, including loss or decrease in appetite, weakness or fatigue, depressed mood, poor coat appearance, and increased water consumption. Increased urination and weight or muscle loss were also significantly associated with advancing CKD stages, reflecting the progressive systemic impact of renal dysfunction. These findings are consistent with previous reports describing polyuria, polydipsia, anorexia and weight loss as among the earliest and most frequent clinical manifestations of feline CKD [3,8,13,22,58]. Overall, the gradual increase in both the frequency and severity of these signs across CKD stages highlights their diagnostic and prognostic significance. Early recognition of subtle changes in appetite, behavior, or water intake may facilitate timely intervention, dietary modification, and improved quality-of-life outcomes for affected cats.

In addition to the clinical prevalence of specific signs, the present study explored how these manifestations were perceived by owners in terms of their impact on both the cat and themselves. For the cat, the pet owners’ answers reflect the key clinical manifestations previously identified as correlating with disease severity. For themselves, the answers reflect their aesthetic or sensory implications in daily interaction. The owners’ perceptions are consistent with recent findings that cats with CKD experience a marked reduction in HRQL, particularly in dimensions related to comfort, vitality, and emotional wellbeing. Moreover, these observations align with recent findings emphasizing the relevance of owner perception and emotional burden in the management of chronic feline diseases [23,35,63]. Caregiver stress and frustration may influence adherence and care quality, highlighting the importance of communication strategies that support both the cat’s welfare and the owner’s wellbeing [23,64]. Our findings show that owners may not recognize the most severe indicators of poor welfare. This suggests the veterinarian’s role in educating owners to shift their focus from ‘cosmetic’ signs to the crucial indicators of wellbeing, such as appetite, interaction, and activity levels.

Our study population was characterized by a predominance of early stage fCKD. At Timepoint 1, 64.9% of the cases were practitioner-assigned as IRIS Stage 1 and 2. This distribution is consistent with population-based or screening studies [7], and suggests that growing awareness and improved diagnostics may result in earlier detection, which is beneficial for disease prognosis and the cats’ QoL [16].

Changes in practitioner-assigned IRIS stages classification over time were observed in 28.8% of the 177 cases with data collected at both Timepoints 1 and 2. Overall, 11.3% of the cases moved to a higher stage, while 17.51% shifted to a lower stage. Advancement to a higher stage is expected in a progressive disease, and it can be attributed to a sudden worsening of the clinical condition. A shift to a lower stage, however, may be attributed to the successful management of pre-renal azotemia (e.g., from dehydration or a UTI), the stabilization of a patient after an acute-on-chronic kidney disease event [1] or even the inherent biological variability of creatinine [65].

The median age of fCKD cases in our study was 10 years old. Although fCKD is traditionally considered a disease of geriatric cats [2], our finding that a substantial proportion of cases included middle-aged animals (7–10 years) [66,67] may also reflect this trend of earlier detection. Moreover, it may be suggestive of the multifactorial nature of fCKD beyond age-related renal decline due to progressive nephron loss and tubulointerstitial fibrosis [1]. This contrasts with some studies reporting higher mean ages [7,68] and supports the view that age alone is not the sole determinant of disease onset or severity. Consistent with the literature, we found no association with sex or breed, suggesting that fCKD is a widespread condition in the cat population [17].

The findings regarding the diet administered to the diseased cats emphasize that a substantial portion of them were not managed strictly with a prescription renal diet. Furthermore, the wide consumption of canned food and pouches indicates that hydration concerns and palatability may influence dietary choices, a critical factor given the risk of food aversion in CKD [18,68]. Despite strong evidence of benefit, long-term adherence to renal diets remains a major clinical challenge, often limited by reduced appetite, food aversion, and difficulties in owner compliance [18,68].

This study has several important limitations inherent to its real-world data design. A primary limitation relates to the integrity of clinical staging. IRIS staging and comorbidity diagnosis were performed by 42 different practitioners, introducing the potential for inter-observer variability in clinical assessment and IRIS staging. This may have contributed to the observed stage migration. The stage shift to lower stages likely reflects not only clinical stabilization but also the variability of practitioner-based staging in a real-world setting. Consequently, the IRIS stages reported should be interpreted as “practitioner-assigned clinical stages” rather than standardized classifications. Future studies employing a centralized review of cases could standardize these variables.

This is further compounded by the lack of SDMA and UP/C data in the overall study population. The lack of uniformly available SDMA data limited the ability to fully assess its contribution to IRIS staging in this real-world study. Seemingly, the lack of uniformly available UP/C data did not allow sub-staging and potentially led to the misclassification of some Stage 1 cats. Financial restrictions that do not allow for assessing a complete panel of renal biomarkers and the challenge of urine collection in cats reflect practical difficulties in a clinical setting. However, future studies should include biomarkers such as SDMA and specialized tools for urine collection in cats.

Another limitation is the lack of standardized diagnostic criteria for the recorded comorbidities. Conditions like anemia and hypertension were reported as present or absent by the practitioners without accompanying quantitative data, such as hematocrit values or specific blood pressure readings. This did not allow the analysis of the association of the severity of these conditions with HRQL. For instance, while our data shows a strong negative association between the presence of anemia and QoL, we cannot differentiate between the impact of mild versus severe anemia. Similarly, diagnoses of infectious diseases such as FIV and FeLV were recorded without accompanying data on the specific testing method or the clinical status of the infection. This limits a more detailed interpretation of how specific pathogens or disease statuses affect HRQL. While the small number of affected cats and their aggregation into a broader ‘Infectious diseases’ category for regression analysis minimizes the statistical impact of this variability, we acknowledge that this lack of detail is a limitation. Future research employing standardized diagnostic protocols would be necessary to clarify these specific relationships.

An additional limitation of this study is the potential for cluster effects related to the 42 different practitioners and clinics contributing data. The study population included cases from both general and referral practitioners. It is expected that different clinics have different protocols or levels of emphasis on diagnostics, such as the frequency of performing dental exams or blood work. This variability could influence the detection rates of certain comorbidities and introduce bias into the observed associations with HRQL. Consequently, the findings should be interpreted with the acknowledgment that some of the observed variation may be attributable to differences between clinics. While we acknowledge that clinic-level cluster effects could influence detection rates, the uneven distribution of cases across 42 practitioners—many contributing only 1–2 cases—precluded the use of multi-level modeling. Such an approach would have risked model instability and compromised the validity of the reported odds ratios.

Additionally, the generalizability of our findings is limited by the sampling strategy. The study utilized a convenience sample of practitioners, which may not be fully representative of all veterinary clinics in Greece. Veterinarians within the AOH network may represent a more engaged or proactive group, which could contribute to the high proportion of early-stage diagnoses. As such, our findings apply to the specific conditions and practices of this cohort, and caution should be taken when generalizing to other countries or healthcare systems.

Finally, we chose to analyze data from the largest possible sample size and not perform a longitudinal analysis on the 177 cats with data from two timepoints. Thus, the cross-sectional nature of our study allows us to interpret our findings on comorbidities and QoL as associations, not as predictors of progression. Future longitudinal analysis of this cohort is warranted to investigate if the highly associated comorbidities identified here are predictive of more rapid stage progression or a faster decline in HRQL scores over time.

## 5. Conclusions

In conclusion, this study demonstrates that while fCKD severity is a key determinant of HRQL, the management of comorbidities is equally critical to a cat’s welfare. Our real-world data demonstrates that a cat with fCKD is not only a ‘kidney patient,’ but a complex case whose QoL is led by their comorbidities and their IRIS stage as well, with our data showing these associations are independent of disease stage. These findings have direct clinical implications for routine practice, emphasizing the importance of a holistic management approach that extends beyond renal function alone. Based on these findings, we recommend that clinicians: (1) integrate formal HRQL assessments into routine management to record the patient’s QoL, (2) screen for and treat highly associated comorbidities, particularly oral/dental disease and anemia, and (3) educate owners to monitor for the most clinically relevant signs of welfare decline, such as changes in appetite and activity. Such an approach may support more targeted clinical decision-making and improved welfare outcomes in cats with fCKD. Future longitudinal research is warranted to determine if targeted interventions against these factors can improve HRQL scores over time.

## Figures and Tables

**Table 1:** Distribution of fCKD cases by IRIS stage as reported by the practitioners (Timepoint 1 and Timepoint 2).

IRIS Stages	Timepoint 1 N (%, 95%CI)	Timepoint 2 N (%, 95%CI)
Stage 1	43 (20.7, 15.2–26.2)	51 (28.8, 22.2–35.4)
Stage 2	92 (44.2, 37.5–51.0)	75 (42.4, 35.1–49.7)
Stage 3	44 (21.2, 15.6–26.7)	34 (19.2, 13.5–24.9)
Stage 4	29 (13.9, 9.2–18.7)	17 (9.6, 5.3–13.9)
Total	208 (100.0)	177 (100.0)

Among the 177 cases with data collected in both Timepoints 1 and 2, the practitioner-assigned IRIS stage remained consistent in 126 cases. Stage migration to a higher IRIS stage at Timepoint 2 was reported in 20 cases (11.3%), and to a lower IRIS stage in 31 cats (17.5%).

**Table 2:** Sex distribution of fCKD cases by IRIS Stage.

Category	Stage 1, N (%)	Stage 2, N (%)	Stage 3, N (%)	Stage 4, N (%)	Total, Ν (%)
Female Intact	0 (0)	1 (1.1)	0 (0)	2 (6.9)	3 (1.4)
Female Spayed	16 (37.2)	41 (44.6)	23 (52.3)	10 (34.5)	90 (43.3)
Male Intact	1 (2.3)	3 (3.3)	3 (6.8)	0 (0)	7 (3.4)
Male Neutered	26 (60.5)	47 (51.1)	18 (40.9)	17 (58.6)	108 (51.9)
Total, Ν (%)	43 (20.7)	92 (44.2)	44 (21.2)	29 (13.9)	208 (100.0)

Percentages in data cells were calculated using the number of cats within each IRIS stage as the denominator; Percentages in total value cells use the overall study population (N = 208).

**Table 3:** Comparison of VetMetrica™ health-related quality of life (HRQL) scores across practitioner-assigned IRIS stages.

Domain	H Stat	*p*-Value	Stage 1 Median	Stage 2 Median	Stage 3 Median	Stage 4 Median	St. Significant Multiple Comparisons
Vitality	44.68	* <0.001	40.42	44.52	35.00	16.09	4-2 (adj. *p* < 0.001) 4-3 (adj. *p* = 0.019) 4-1 (adj. *p* < 0.001) 3-2 (adj. *p* = 0.006)
Comfort	36.50	* <0.001	38.38	37.08	31.92	25.61	4-1 (adj. *p* < 0.001) 4-2 (adj. *p* < 0.001) 3-2 (adj. *p* = 0.003)
Emotional Wellbeing	45.32	* <0.001	33.09	44.13	27.36	6.88	4-1 (adj. *p* < 0.001) 4-2 (adj. *p* < 0.001) 4-3 (adj. *p* = 0.011) 3-2 (adj. *p* = 0.003)
Physical Wellbeing	45.69	* <0.001	39.02	40.28	33.03	20.63	4-1 (adj. *p* < 0.001) 4-2 (adj. *p* < 0.001) 4-3 (adj. *p* = 0.026) 3-2 (adj. *p* = 0.002)

The statistically significant results are depicted with an asterisk.

**Table 4:** Comparisons of the various VetMetrica scores for early and late IRIS Stages.

Domain	U Stat	*p*-Value	Stage 1–2 Median	Stage 3–4 Median
Vitality	2448	* <0.001	43.52	25.13
Comfort	2596.5	* <0.001	36.77	30.00
Emotional Wellbeing	2584.5	* <0.001	41.21	28.16
Physical Wellbeing	2398	* <0.001	39.44	28.16

The statistically significant results are depicted with an asterisk.

**Table 5:** Comparisons of the various VetMetrica Scores related to the existence or non-existence of concomitant diseases.

Domain	U Stat	*p*-Value	No Concomitant Median	With Concomitant Median
Vitality	2863	* <0.001	48.26	35.97
Comfort	2801	* <0.001	39.96	32.56
Emotional Wellbeing	2360	* <0.001	48.83	23.86
Physical Wellbeing	2700	* <0.001	46.25	32.70

The statistically significant results are depicted with an asterisk.

**Table 6:** Odds Ratios for VetMetrica HRQL domain deterioration by comorbidity category in cats with fCKD.

Category of Comorbidities	Domain Score	OR	95%CI	*p*-Value
Hematologic	Vitality	7.0	1.6–30.3	* 0.009
Oral/Dental	Vitality	2.6	1.2–5.5	* 0.016
Hematologic (anemia)	Comfort	14.9	2.0–111.5	* 0.009
Oral/Dental	Comfort	5.2	2.1–12.8	* <0.001
Hematologic	Emotional Wellbeing	12.7	1.7–95.1	* 0.014
Oral/Dental	Emotional Wellbeing	7.1	2.4–20.7	* <0.001
Hematologic	Physical Wellbeing	13.0	1.7–97.8	* 0.013
Oral/Dental	Physical Wellbeing	4.4	1.8–11.0	* 0.001

Note: The statistically significant results are depicted with an asterisk.

**Table 7:** Results of multiple regression analysis for the association of specific comorbidities with HRQL domain scores.

Domain Score	Comorbidity	Expected Domain Score When No Comorbidity	Deterioration in the Domain Score	SD±	*p*-Value
Vitality	Hematologic diseases (anemia)	41.64 (±1.68)	−15.00	3.19	* <0.001
	Oral diseases		−5.78	2.55	* 0.025
Comfort	Hematologic diseases (anemia)	40.98 (±1.06)	−7.99	2.01	* <0.001
	Oral diseases		−5.09	1.61	* 0.002
	Infectious diseases		−7.77	2.36	* 0.001
Emotional Wellbeing	Hematologic diseases (anemia)	38.95 (±1.87)	−18.79	3.55	* <0.001
	Oral diseases		−7.25	2.85	* 0.012
	Infectious diseases		−9.34	4.12	* 0.026
Physical Wellbeing	Hematologic diseases (anemia)	41.30 (±1.28)	−11.50	2.43	* <0.001
	Oral diseases		−5.436	1.94	* 0.006
	Infectious diseases		−6.110	2.845	* 0.033

The statistically significant results are depicted with an asterisk.

## Data Availability

The data presented in this study are available on request from the corresponding author. The datasets for this article are not publicly available due to concerns regarding owner/practitioner anonymity.

## Supplementary Materials

The following supporting information can be downloaded at: https://www.mdpi.com/article/10.3390/vetsci13020192/s1, Figure S1: Histogram and kernel density estimation (KDE) for feline age, showing the Free Probability Density Function (PDF) for the normal distribution; Table S1: Eligibility criteria for observational study; Table S2: Description of the International Renal Interest Society (IRIS) staging guidelines in cats; Table S3: Veterinarian-completed form; Table S4: Case sheet of cat with chronic kidney disease: Animal diet, disease history and treatment plan; Table S5: Case sheet of cat with chronic kidney disease: Clinical signs and most troublesome clinical signs for the cat and the cat owner; Table S6: Owner-completed form; Table S7: Owners’ demographics; Table S8: Categorization of reported concomitant diseases; Table S9: Feline breeds by IRIS Stage; Table S10: Clinical signs of cats with fCKD, as reported by the practitioners; Table S11: Point-biserial correlation between presence of clinical signs and severity of fCKD according to IRIS Stage; Table S12: Clinical signs reported by the owner as more troublesome for the owner and the cat; Table S13: Descriptive statistics of the biochemical examinations performed in the study population; Table S14: Comparison of analyte concentrations across IRIS stages using ANOVA and Kruskal-Wallis tests; Table S15: Urine examination results; Table S16: Prevalence of Concomitant Disease Categories and 95% Confidence Intervals; Table S17: List of Comorbidities reported in fCKD cases; Table S18: Results of Permutational Multivariate Analysis of Variance (PERMANOVA) for Concomitant Conditions and combined QoL measures.

## Abbreviations

The following abbreviations are used in this manuscript:

ALB	Albumin
AOH	Asclepius One Health
BCS	Body Condition Score
BUN	Blood urea nitrogen
Ca and Ca++	Calcium
CKD	Chronic kidney disease
Cl	Chloride
CREA	Blood creatinine
DSH	Domestic Shorthair
fCKD	Feline chronic kidney disease
HRQL	Health-related quality of life
IQR	Interquartile Range
IRIS	International Renal Interest Society
K	Potassium
MCS	Muscle Condition Score
Na	Sodium
OR	Odds ratios
P	Phosphorus
PERMANOVA	Permutational Multivariate Analysis of Variance
QoL	Quality of life
RWD	Real-world data
RWE	Real-world evidence
r	Pearson correlation coefficient
rpb	Point-biserial correlation coefficient
SDMA	Symmetric dimethylarginine
SG	Specific gravity
sCr	Serum creatinine
TP	Total Proteins
UP/C	Urine protein-to-creatinine
