# Supplementary material for: A Real-World Data Analysis on Feline Chronic Kidney Disease in Greece: Clinical Profiles, Comorbidities, and Quality of Life
Source: Vet Sci. 2026 Feb 15;13(2):192. doi: 10.3390/vetsci13020192 (PMC12945146; doi:10.3390/vetsci13020192)
Supplement: Supplementary file 1 [file vetsci-13-00192-s001.zip › vetsci-4123627-supplementary.pdf]

veterinary sciences

MDPI

Supplementary Materials

Table S1. Eligibility criteria for observational study

Inclusion criteria	The cat must have a veterinarian-confirmed diagnosis of CKD with any IRIS stage (i.e., Stage 1, 2, 3, or 4).<br>The cat must be stable and been treated for, or is currently being treated for, CKD. If stable and staged up to three months prior to recruitment, staging bloodwork is to be performed and the cat should stabilize before the owner completes the instruments.<br>The cat must be a minimum of one year of age.<br>The owner of the cat with CKD must be 18 years or above and the primary caretaker.<br>The owner of the cat with CKD is willing and able to answer written questions and instruments in Greek related to their cat with CKD at two different timepoints.
Exclusion criteria	The cat is known or suspected to be pregnant or lactating.<br>The cat is eating an unbalanced diet.<br>The cat required medical hospitalization in the seven days immediately prior to the owner completed questionnaires.<br>The cat has any of the following medical conditions:<br>Heartworm disease<br>Uncontrolled hyperparathyroidism<br>Primary hyperparathyroidism or primary hypoparathyroidism<br>Protein-losing enteropathy<br>Polycystic kidney disease, renal dysplasia<br>Hypercalcemia of renal or non-renal secondary hyper parathyroid origin<br>Pyelonephritis currently, hospitalized with a diagnosis of pyelonephritis (however, if cats with episodes longer than one year ago that have resolved, these cats can participate).<br>Post-renal azotemia such as obstructive lower urinary tract disease/urolithiasis (felines with episodes longer than one year in the past are not excluded).<br>Any malignant or suspected malignant neoplasia.<br>Acute Kidney Injury (AKI) or acute azotemia. For example, do not enroll felines in uremic crisis (ethylene glycol toxicity, acute on chronic episodes,

	blocked cat) but once stable this feline can be enrolled in study at a future time when at a stable IRIS stage.<br><br>The cat is receiving treatment, or a history of requiring treatment in the last six months, for active infectious diseases that secondarily causes nephropathology, such as Lyme nephritis, ehrlichiosis, leishmaniosis or leptospirosis.<br><br>The cat is enrolled in a clinical study in which an investigational veterinary product is used.

Table S2. Description of the International Renal Interest Society (IRIS) staging guidelines in cats

	Stage 1: Blood Creatinine <140 µmol/l or <1.6 mg/dl, SDMA <18 µg/dl.<br>Normal blood creatinine or normal or mild increase blood SDMA. Some other renal abnormality present (such as inadequate urinary concentrating ability without identifiable non-renal cause (in cats not cats), abnormal renal palpation or renal imaging findings, proteinuria of renal origin, abnormal renal biopsy results, increasing blood creatinine or SDMA concentrations in samples collected serially). Persistently elevated blood SDMA concentration (>14 µg/dl) may be used to diagnose early CKD.
Description of IRIS Stages	Stage 2: Blood Creatinine 140-250 µmol/l or 1.6-2.8 mg/dl, SDMA 18-25 µg/dl.<br>Normal or mildly increased creatinine, mild renal azotemia (lower end of the range lies within reference ranges for creatinine for many laboratories, but the insensitivity of creatinine concentration as a screening test means that patients with creatinine values close to the upper reference limit often have excretory failure). Mildly increased SDMA. Clinical signs usually mild or absent.
	Stage 3: Blood Creatinine 251-440 µmol/l or 2.9-5.0 mg/dl, SDMA 26-38 µg/dl.<br>Moderate renal azotemia. Many extrarenal signs may be present, but their extent and severity may vary. If signs are absent, the case could be considered as early Stage 3, while presence of many or marked systemic signs might justify classification as late Stage 3.
	Stage 4: Blood Creatinine >440 µmol/l or >5.0 mg/dl, SDMA >38 µg/dl.<br>Increasing risk of systemic clinical signs and uremic crises.

Table S3. Veterinarian-completed form

Information	Question in full	Response options
Age	What is the cat's age?	Free text
Sex	What is the cat's sex?	Male intact<br>Female intact<br>Male neutered<br>Female spayed
Breed	What is the cat's breed?	Free text
Weight	What is the cat's weight?	Free text

BCS	From 1-9, choose the cat's BCS	1-9
MCS	From A-D, choose the cat's MCS	A-D
Comorbidities	Indicate any diagnosed concomitant conditions for this cat	Hypertrophic cardiomyopathy<br>Congestive heart failure<br>Peripheral vascular disease<br>Cerebrovascular disease<br>Hyperthyroidism<br>Hemiplegia or paraplegia<br>Dementia<br>Degenerative Joint Disease/Arthritis<br>Stomach and intestinal ulcers<br>Diabetes<br>Any malignancy, including leukemia and lymphoma<br>Metastatic solid tumor<br>Mild liver disease<br>Moderate or severe liver disease<br>Hypertension<br>Anemia<br>Heart Murmur<br>Hypercalcemia<br>Periodontal disease<br>Cystitis<br>Urolithiasis<br>Cardiac abnormalities<br>Pathogenic infection<br>Obesity<br>Glomerulonephritis<br>Dermatitis

	Atopy
	Stomatitis
	Feline Immunodeficiency Virus (FIV)
	Feline asthma
	Megacolon
	Constipation
	Chronic Upper Respiratory Tract Disease
	Other (specify)__________
	No concomitant conditions

Table S4. Case sheet of cat with chronic kidney disease: Animal diet, disease history and treatment plan

Information	Question in full	Response options
Cat info	Cat name	Free text
Cat info	Microchip number	Free text
Animal diet	Record basic diet information (cooked foods, commercial, clinical diets, etc.)	Free text
Disease history	Record the animal's basic medical history that may be related to CKD	Free text
Treatment plan	Record any medications (active substance) the cat is currently being administered, as well as the dose regimen	Free text

Table S5. Case sheet of cat with chronic kidney disease: Clinical signs and most troublesome clinical signs for the cat and the cat owner.

	**Clinical signs currently present**	**Most troublesome clinical signs**	
		**For your cat**	**For you, when caring for your cat**
1. Increased/excessive water consumption	□	□	□
2.Increased/excessive urination	□	□	□
3. Unexplained weight loss / loss of muscle mass / body condition	□	□	□
4. Loss/decrease of appetite	□	□	□

5. Anorexia	□	□	□
6. Depressed mood	□	□	□
7. Weakness/fatigue	□	□	□
8. Vomiting	□	□	□
9. Diarrhoea	□	□	□
10. Halitosis / Very bad breath	□	□	□
11. Pale gums	□	□	□
12. Stomach / intestinal inflammation (gastroenteritis)	□	□	□
13. Mouth ulcers	□	□	□
14. Problems with vision	□	□	□
15. Unkept Appearance	□	□	□
16. Fragile bones	□	□	□
17. Blood in urine	□	□	□
18. Other (please specify) ________	□	□	□
19. Other (please specify) ________	□	□	□
20. My cat has/had no clinical signs	□	□	□

Table S6. Owner-completed form

Demographics	Question in full	Response options
Gender	Are you:	Male; Female; Intersex
Age	What is your age?	Free text
Employment	What is your current main employment status?	I work full time<br>I work part time<br>I'm a student<br>I'm not working because I choose not to<br>I'm unemployed<br>I'm not working because I am retired<br>I'm on long term sick leave
Education	What is your highest level of education?	Primary education (e.g. primary school<br>Secondary education (e.g. Gymnasium or Lyceum)

	College or Associate degree (vocational or academic)<br>Undergraduate degree (Bachelor's degree, Professional degree or equivalent)<br>Graduate degree (Masters, Doctorate or equivalent)<br>Not listed, please specify<br>_______________

Table S7. Owners' demographics

Characteristic	Category	Male	Female	Total
**Age Statistics**	Mean Age (years)	46.4	45.7	
	Median Age (years)	45.0	45	
	Standard Deviation	12.8	13.2	
	Age Range (years)	20 - 74	20 - 78	
**Employment Status**	I work full time	23 (69.7)	93 (53.4)	116 (56.0)
	I work part time	1 (3.0)	34 (19.5)	35 (16.9)
	I'm a student	2 (6.1)	2 (1.1)	4 (1.9)
	I'm not working because I am retired	2 (6.1)	15 (8.6)	17 (8.2)
	I'm unemployed	1 (3.0)	11 (6.3)	12 (5.8)
	I'm not working because I am retired	4 (12.1)	19 (10.9)	23 (11.1)
	Primary education	0 (0.0)	1 (0.6)	1 (0.5)
	Secondary education	5 (15.2)	30 (17.2)	35 (16.9)
	College or Associate degree	12 (36.4)	33 (19.0)	45 (21.7)
	Undergraduate degree	15 (45.5)	77 (44.3)	92 (44.4)
	Graduate degree	1 (3.0)	33 (19)	34 (16.4)
**Total Respondents**		33 (15.9)	174 (84.1)*	207 (100.0)

*One owner refused to answer the questions about employment and education. Percentages for male and female categories were calculated using the total number of respondents within each gender group as the denominator (N=33 for Male, N=174 for Female).

Table S8. Categorization of reported concomitant diseases

Cardiovascular	Congestive heart failure, Peripheral vascular disease, Hypertension, Heart Murmur, Cardiac abnormalities, Hypertrophic cardiomyopathy
Musculoskeletal	Degenerative Joint Disease/Arthritis, Amputation of left forelimb
Neurological diseases	Cerebrovascular disease, Hemiplegia or paraplegia, Dementia, Idiopathic Epileptic Seizures
Hematologic diseases	Anemia
Renal/Urinary diseases	Cystitis, Urolithiasis, Glomerulonephritis, Bilateral subcapsular kidney cysts

Gastrointestinal diseases	Stomach and intestinal ulcers, Megacolon, Constipation, Intestinal Bowel Disease (IBD), Triaditis
Cancer	Any malignancy, including leukemia and lymphoma, Metastatic solid tumor
Dermatologic	Dermatitis, Atopy
Endocrine/metabolic diseases	Diabetes, Hypercalcaemia, Hyperthyroidism
Liver diseases	Mild liver disease, Moderate or severe liver disease
Oral/Dental diseases	Periodontal disease, Stomatitis
Infectious diseases	Pathogenic infection, Feline Immunodeficiency Virus (FIV), Feline Infectious Peritonitis (FIP), Feline Leukemia Virus (FeLV), Hemobartonellosis, Purulent rhinotracheitis, Pyothorax (due to catfight)
Respiratory diseases	Feline asthma, Chronic Upper Respiratory Tract Disease
Ocular diseases	Blindness, Retinal Degeneration
Obesity	Obesity
Other	Diarrhea, Epiphora

Table S9. Feline breeds by IRIS Stage

Breed	Stage1 N (%)	Stage 2 N (%)	Stage 3 N (%)	Stage 4 N (%)	Total N (%)
Domestic Shorthair (DSH)	36 (21.7)	71 (42.8)	35 (21.1)	24 (14.5)	166 (79.8)
Norwegian Forest Cat	0 (0)	1 (100)	0 (0)	0 (0)	1 (0.5)
British Shorthair	0 (0)	0 (0)	0 (0)	1 (100)	1(0.5)
European Longhair	5 (38.5)	4 (30.8)	2 (15.4)	2 (15.4)	13 (6.3)
Siamese	1 (33.3)	2 (66.7)	0 (0)	0 (0)	3 (1.4)
Persian	0 (0)	7 (58.3)	5 (41.7)	0 (0)	12 (5.8)
European Shorthair	1 (9.1)	6 (54.5)	2 (18.2)	2 (18.2)	11 (5.3)
Scottish Straight	0 (0)	1 (100)	0 (0)	0 (0)	1(0.5)

Percentages within each IRIS stage column represent the proportion of that breed found in that stage. The "Total" column indicate the prevalence of each breed within the overall study population (N = 208).

Table S10. Clinical signs of cats with fCKD, as reported by the practitioners.

Clinical signs reported	Stage 1 N	Stage 1 %	Stage 2 N	Stage 2 %	Stage 3 N	Stage 3 %	Stage 4 N	Stage 4 %	Total N	Total %
Increased/excessive water consumption	17	39.5	40	43.5	22	50.0	26	89.7	105	50.5
Increased/excessive urination	14	32.6	31	33.7	15	34.0	24	82.8	84	40.4
Unexplained weight loss / loss of muscle mass / body condition	16	37.2	31	33.7	18	40.9	20	69.0	85	40.9
Loss/decrease of appetite	7	16.3	26	28.3	21	47.7	26	89.7	80	38.5
Anorexia	2	4.7	1	1.1	3	6.8	6	20.7	12	5.8
Depressed mood	5	11.6	9	9.8	9	20.5	15	51.7	38	18.3

Weakness/fatigue	3	7.0	13	14.1	13	29.5	16	55.2	45	21.6
Vomiting	14	32.6	17	18.5	11	25	13	44.8	55	26.4
Diarrhoea	4	9.3	6	6.5	1	2.3	0	0.0	11	5.3
Halitosis / Very bad breath	9	20.9	24	26.3	16	36.4	12	41.4	61	29.3
Pale gums	4	9.3	4	4.3	8	18.2	8	27.6	24	11.5
Stomach / intestinal inflammation (gastroenteritis)	2	4.7	3	3.3	1	2.3	2	6.9	8	3.8
Mouth ulcers	2	4.7	4	4.3	2	4.5	5	17.2	13	6.3
Vision disorders	1	2.33	4	4.3	2	4.5	0	0.0	7	3.4
Unkept appearance	8	18.6	23	25.0	15	34.1	18	62.1	64	30.8
Fragile bones	0	0.0	1	1.1	0	0.0	0	0.0	1	0.5
Blood in urine	2	4.7	2	2.2	2	4.5	2	6.9	8	3.8
Total	43	20.7	91	44.2	44	21.2	29	13.9	208	100.0

Percentages were calculated using the number of cats within each IRIS stage as the denominator; Total values use the overall study population.

Table S11. Point-biserial correlation between presence of clinical signs and severity of fCKD according to IRIS Stage. The statistically significant results are depicted with an asterisk.

Clinical signs reported	Correlation coefficient	p-value
Increased/excessive water consumption	0.277	<0.001*
Increased/excessive urination	0.261	<0.001*
Unexplained weight loss / loss of muscle mass / body condition	0.185	0.008*
Loss/decrease of appetite	0.452	<0.001*
Anorexia	0.209	0.002*
Depressed mood	0.305	<0.001*
Weakness/fatigue	0.361	<0.001*
Vomiting	0.085	0.221
Diarrhoea	-0.139	0.045*
Halitosis / Very bad breath	0.153	0.028*
Pale gums	0.210	0.002*
Stomach / intestinal inflammation (gastroenteritis)	0.019	0.782
Mouth ulcers	0.132	0.056
Vision disorders	-0.028	0.691
Poor coat condition	0.273	<0.001*
Fragile bones	-0.021	0.765
Blood in urine	0.046	0.512

The statistically significant results are depicted with an asterisk.

Table S12. Clinical signs reported by the owner as more troublesome for the owner and the cat.

Clinical signs	Troublesome for the Cat n (% [95% CI])	Troublesome for the Owner n (% [95% CI])	Total Cases (N)	Fischer's p-value
Increased/excessive water consumption	48 (85.7% [76.5, 94.9])	20 (35.7% [23.2, 48.3])	56	0.002*
Increased/excessive urination	37 (67.3% [54.9, 79.7])	35 (63.6% [50.9, 76.3])	55	<0.001*
Unexplained weight loss / muscle mass	39 (69.6% [57.6, 81.7])	31 (55.4% [42.3, 68.4])	56	<0.001*
Loss/decrease of appetite	39 (70.9% [58.9, 82.9])	35 (63.6% [50.9, 76.3])	55	<0.001*
Anorexia	7 (77.8%)	6 (66.7%)	9	-
Depressed mood	20 (83.3% [69.6, 97.0])	10 (41.7% [20.0, 63.3])	24	0.020*
Weakness/fatigue	20 (83.3% [69.6, 97.0])	15 (62.5% [40.9, 84.1])	24	0.259
Vomiting	31 (86.1% [73.5, 98.7])	23 (63.9% [49.2, 78.6])	36	0.136
Diarrhoea	9 (90.0%)	8 (80.0%)	10	-
Halitosis / Very bad breath	19 (47.5% [32.9, 62.1])	34 (85.0% [72.6, 97.4])	40	0.007*
Pale gums	5 (83.3%)	2 (33.3%)	6	-
Stomach/intestinal inflammation	5 (83.3%)	4 (66.7%)	6	-
Mouth ulcers	7 (100.0%)	1 (14.3%)	7	-
Vision disorders	6 (100.0%)	3 (50.0%)	6	-
Unkept Appearance	20 (62.5% [45.9, 79.1])	21 (65.6% [48.9, 82.3])	32	0.002*
Fragile bones	-	-	-	-
Blood in urine	2 (50.0%)	2 (50.0%)	4	-

*Statistically significant results. Confidence intervals and statistical tests were conducted only for clinical signs with at least 20 cases.

Table S13. Descriptive statistics of the biochemical examinations performed in the study population.

Biochemical Parameter	N	Median	IQR (25th – 75th Percentile)	Minimum	Maximum
TP	207	8.80	[8.00 – 9.55]	5.50	13.20
CREA	208	2.12	[1.68 – 3.48]	0.73	16.64
ALB	208	3.00	[2.70 – 3.20]	1.80	3.90
Ca	207	9.50	[9.00 – 10.30]	7.40	14.90

Ca++	206	1.21	[1.17 – 1.32]	0.93	1.55
K	205	4.40	[4.00 – 4.90]	2.90	7.70
Na	205	153.00	[148.00 – 155.00]	132.00	162.00
Cl	206	113.00	[109.00 – 117.00]	76.00	125.00
P	208	5.05	[4.25 – 6.40]	2.30	28.20
BUN	208	43.00	[31.00 – 78.50]	14.00	319.00
Na/K	205	34.44	[31.04 – 37.75]	17.47	55.17

Table S14. Comparison of analyte concentrations across IRIS stages using ANOVA and Kruskal-Wallis tests.

Variable	F-statistic	ANOVA p-value	Adjusted ANOVA p-value (BH)	Kruskal-Wallis χ^2	Kruskal-Wallis p-value	Adjusted KW p-value (BH)
CREA	-	-	-	184.45	< .001	< .001*
BUN	-	-	-	146.77	< .001	< .001*
P	-	-	-	73.08	< .001	< .001*
ALB	0.67	0.571	0.628	-	-	-
Cl	-	-	-	7.47	0.058	0.160
K	-	-	-	6.49	0.090	0.198
TP	-	-	-	6.37	0.095	0.198
Na/K	-	-	-	5.00	0.172	0.270
Ca++	-	-	-	3.85	0.278	0.382
Ca	-	-	-	2.46	0.483	0.590
Na	-	-	-	1.02	0.796	0.796

Significance is determined at $p < 0.05$. For each variable, results from either ANOVA or the non-parametric Kruskal-Wallis (KW) test are shown. P-values were adjusted for multiple comparisons using the Benjamini-Hochberg (BH) method. Significant adjusted p-values are shown with an asterisk.

Table S15. Urine examination results.

Statistic	SG	PH	UP/C
**Count**	35	35	35

**Mean**	1023.77	6.66	0.66
**Standard Deviation**	10.16	0.73	0.69
**Minimum**	1010.0	5.5	0.06
**Median**	1020.0	6.5	0.41
**Maximum**	1045.0	8.0	2.62

Table S16. Prevalence of Concomitant Disease Categories and 95% Confidence Intervals.

**Category**	**N**	**%**	**95% CI**
Cardiovascular	20	9.6	[5.5, 13.8]
No concomitant conditions	69	33.2	[26.9, 39.5]
Musculoskeletal	10	4.8	[1.9, 7.8]
Neurological	4	1.9	[0.1, 3.8]
Hematologic (anemia)	32	15.4	[10.3, 20.5]
Renal disease/Urinary Tract	20	9.6	[5.5, 13.8]
Gastrointestinal	10	4.8	[1.9, 7.8]
Dermatologic	10	4.8	[1.9, 7.8]
Endocrine/metabolic	7	3.4	[0.9, 5.8]
Liver	18	8.7	[4.8, 12.6]
Oral/Dental	62	29.8	[23.5, 36.1]
Infectious	22	10.6	[6.4, 14.8]
Respiratory	16	7.7	[4.0, 11.4]
Ocular	2	1.0	[0.0, 2.3]
Obesity	4	1.9	[0.1, 3.8]
Other	2	1.0	[0.0, 2.3]

Table S17. List of Comorbidities reported in fCKD cases

**Comorbidity**	**N**	**%**
No comorbidities	69	33.2
Periodontal disease	46	22.1
Stomatitis	35	16.8
Anemia	32	15.4
Mild liver disease	15	7.2
Feline immunodeficiency virus (FIV)	14	6.7
Chronic upper respiratory tract disease	13	6.3
Heart murmur	10	4.8
Degenerative joint disease/arthritis	10	4.8

Constipation	8	3.9
Hypertrophic cardiomyopathy	7	3.4
Urolithiasis	7	3.4
Cystitis	7	3.4
Hypertension	7	3.4
Glomerulonephritis	6	2.9
Dermatitis	6	2.9
Atopy	6	2.9
Hyperthyroidism	6	2.9
Feline Infectious Peritonitis (FIP)	5	2.4
Asthma	4	1.9
Obesity	4	1.9
Feline Leukemia Virus (FeLV)	4	1.9
Moderate or severe liver disease	3	1.4
Dementia	3	1.4
Pathogenic infection	2	1.0
Congestive heart failure	2	1.0
Hemobartonellosis	2	1.0
Cardiac abnormalities	1	0.5
Epiphora	1	0.5
Retinal Degeneration	1	0.5
Blindness	1	0.5
Hypercalcemia	1	0.5
Stomach and intestinal ulcers	1	0.5
Diarrhea	1	0.5
Megacolon	1	0.5
Idiopathic epilepsy	1	0.5
Peripheral vascular disease	1	0.5
Triaditis	1	0.5
Bilateral subcapsular kidney cysts	1	0.5
Amputation of left forelimb	1	0.5

Table S18. Results of Permutational Multivariate Analysis of Variance (PERMANOVA) for Concomitant Conditions and combined QoL measures.

Source of Variation	Df	Sum of Sqs	R^2	F	p-value
Concomitant Conditions	1	21,828	0.130	30.908	0.001*
Residual	206	145,483	0.870		
Total	207	167,311	1.000		

The statistically significant results are depicted with an asterisk.

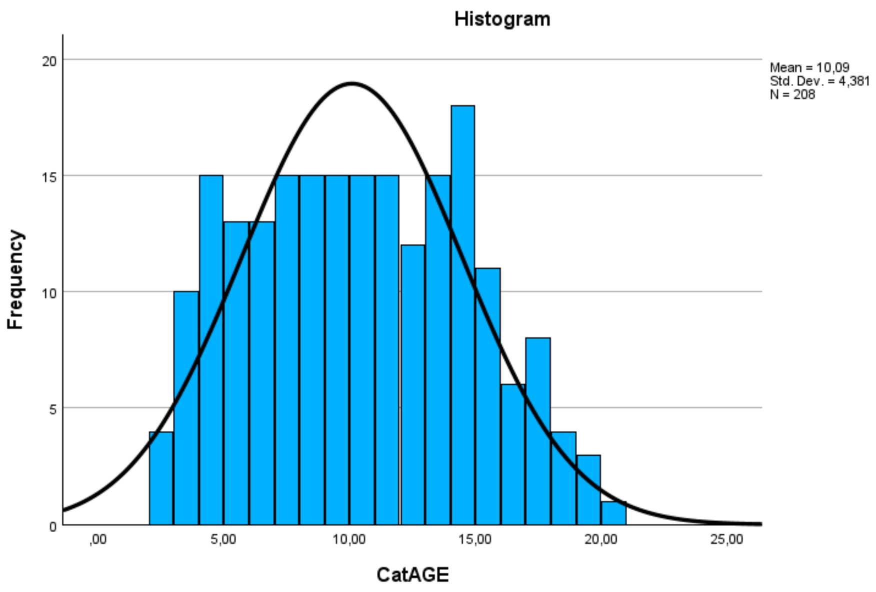

Figure S1. Histogram and kernel density estimation (KDE) for feline age, showing the Free Probability Density Function (PDF) for the normal distribution.
